# Glycerine Cement

**Published:** 1880-08

**Authors:** 


Glycerine Cement.—According to *Dingler's Journal*, Professor Morawski finds that the cause of the hardening of glycerine cement is the formation of lead glyceride, a salt glycerine and lead oxide. This crystallizes in fine needles, and is formed according to the following equation:

$$C_3H_8O_3XPbO=C_3H_6PbOXH_3O.$$

The greatest hardness is obtained by adding 5 cc. of glycerine to 50 grammes of litharge. If more glycerine is used the mass solidifies more slowly, and never gets so hard. To obtain a quickly-hardening cement, two volumes of water should be added to five volumes of glycerine, and 6 cc. of the mixture incorporated with 50 grammes of litharge.

